# Supplementary material for: Understanding quantum machine learning also requires rethinking generalization
Source: Nat Commun. 2024 Mar 13;15:2277. doi: 10.1038/s41467-024-45882-z (PMC10938005; doi:10.1038/s41467-024-45882-z)
Supplement: Supplementary file 1 — Supplementary Information [file 41467_2024_45882_MOESM1_ESM.pdf]

Supplementary Information for "Understanding quantum machine learning also requires rethinking generalization"

SUPPLEMENTARY NOTE 1 - PROOF OF THEOREM 1

In this section, we re-state and prove Theorem 1.

Theorem 1 (Finite sample expressivity of quantum circuits)**.** Let $\rho_1, \dots, \rho_N$ be unknown quantum states on $n \in \mathbb{N}$ qubits, with $N \in \mathcal{O}(\mathrm{poly}(n))$, and let W be the Gram matrix

$$[W]_{i,j} = \mathrm{tr}(\rho_i \rho_j)\,. \tag{1}$$

If W is well-conditioned, then, for any $y_1, \dots, y_N \in \mathbb{R}$ real numbers, we can construct a quantum circuit of depth $\mathrm{poly}(n)$ as an observable $\mathcal{M}_y$ such that

$$\mathrm{tr}(\rho_i \mathcal{M}_y) = y_i\,. \tag{2}$$

Proof. We prove the statement directly and constructively. It is interesting to note that if we had imposed pairwise orthonormal states instead of just linearly independent, the Gram matrix would simply be the identity $W = \mathbb{I}$, in which case we would only need to set

$$\tilde{\mathcal{M}}_y \coloneqq \sum_{k=1}^{N} y_k \rho_k. \tag{3}$$

We would have, for each $i \in \{1, \dots, N\}$,

$$\mathrm{tr}(\rho_i \tilde{\mathcal{M}}_y) = \mathrm{tr}\left(\rho_i \sum_{k=1}^{N} y_k \rho_k\right) = \sum_{k=1}^{N} y_k\, \mathrm{tr}(\rho_i \rho_k) \tag{4}$$

$$= \sum_{k=1}^{N} y_k \delta_{i,k} = y_i\,. \tag{5}$$

In the second-to-last step, we used the pairwise orthogonality and normalization of the quantum states, which we briefly introduced for illustration purposes.

Now, if we go back to only requiring a well-conditioned Gram matrix, we need to introduce the intermediate variable $z = (z_1, \dots, z_N)$, which we define as the solution to the linear system of equations

$$Wz = y\,. \tag{6}$$

We know we can solve this system of linear equations, reaching a unique solution, because W is well-conditioned per hypothesis. With this, we take the same observable as before, but this time with the intermediate variable weighting the sum over states, to get

$$\mathcal{M}_y = \tilde{\mathcal{M}}_z \coloneqq \sum_{k=1}^{N} z_k \rho_k\,. \tag{7}$$

Indeed, this observable produces the correct output for each $i \in [N]$,

$$\mathrm{tr}(\rho_i \tilde{\mathcal{M}}_z) = \mathrm{tr}\left(\rho_i \sum_{k=1}^{N} z_k \rho_k\right) = \sum_{k=1} z_k\, \mathrm{tr}(\rho_i \rho_k) \tag{8}$$

$$= \sum_{k=1}^{N} z_k W_{i,k} = y_i\,. \tag{9}$$

In order to compute $\mathrm{tr}(\rho_i \rho_k)$ for unknown states ρ_i and ρ_k, we could employ the SWAP test, using one auxiliary qubit.

Notice that even though this construction is theoretical and assumes access to many copies of the input states every time we want to run the quantum circuit, the observable's expected outcome can still be estimated in practice following these steps:

1. From y and W, obtain z.
2. From z, obtain the probability distribution $p = (p_1, \dots, p_N)$ as
$$p_k = \frac{|z_k|}{\sum_{j=1}^{N}|z_j|}\,, \tag{10}$$
and the vector of signs $s = (s_1, \dots, s_N)$ as
$$s_k = \frac{|z_k|}{z_k}\,, \tag{11}$$
so that $z_k = s_k p_k \sum_{j=1}^{N}|z_j|$.
3. Sample $k \sim (p_1, \dots, p_N)$ and prepare ρ_k.
4. Estimate $\mathrm{tr}(\rho_i \rho_k)$ for the desired ρ_i and the ρ_k just sampled, for instance using the SWAP test.
5. If $s_k = -1$, flip the outcome in the last step.
6. Repeat the last three steps until convergence.
7. Output the expected value multiplied with $\sum_{j=1}^{N}|z_j|$.

This procedure realizes an unbiased estimator for the expectation value of $\tilde{\mathcal{M}}_z$ with error decreasing linearly with the number of repetitions. With this, the proof is complete.

SUPPLEMENTARY NOTE 2 - PROOF OF THEOREM 2

Here, we re-state and prove Theorem 2.

Theorem 2 (Finite sample expressivity of PQCs)**.** Let $\rho_1, \dots, \rho_N$ be unknown quantum states on $n \in \mathbb{N}$ qubits, with $N \in \mathcal{O}(\mathrm{poly}(n))$, and fulfilling the distinguishability condition of Definition 1. Then, we can construct a PQC of $\mathrm{poly}(n)$ depth as a parametrized observable $\hat{\mathcal{M}}(\vartheta)$ such that, for any $y = (y_1, \dots, y_N) \in \mathbb{R}$ real numbers, we can efficiently find a specification of the parameters ϑ_y such that
$$\mathrm{tr}(\rho_i \hat{\mathcal{M}}(\vartheta_y)) = y_i\,. \tag{12}$$

Proof. This proof follows the steps of Theorem 1. We show the statement directly and constructively. Now, instead of using the quantum states themselves, we shall first find easy-to-prepare approximations and then define the observable based on the latter.

By construction, $\rho_1, \dots, \rho_N$ fulfill the distinguishability condition. That means we can obtain efficient PQC-based approximations $\hat{\rho}_1, \dots, \hat{\rho}_N$, with which we can furbish the matrix $\hat{W}$, with entries
$$[\hat{W}]_{i,j} = \mathrm{tr}(\rho_i \hat{\rho}_j)\,. \tag{13}$$

Moreover, we can efficiently prepare $\hat{\rho}_j$ by applying a now-known parametrized unitary, U_j, to, e.g., the $|0\rangle$ state vector,
$$\hat{\rho}_j = U_j |0\rangle\langle 0| U_j^\dagger\,. \tag{14}$$

This means we do not require using the SWAP test every time anymore, nor continuous coherent access to the input quantum states, since we can now apply the inverse unitary to ρ_i and then measure in the computational basis, to get
$$\mathrm{tr}(\rho_i \hat{\rho}_j) = \mathrm{tr}\left(\rho_i U_j |0\rangle\langle 0| U_j^\dagger\right) = \langle 0| U_j^\dagger \rho_i U_j |0\rangle\,. \tag{15}$$

In the case of ρ_i being a pure state $\rho_i = |\psi_i\rangle\langle\psi_i|$, the inner product is just $|\langle 0| U_j^\dagger |\psi_i\rangle|^2$. This can be clearly computed as a PQC.

We repeat this approach for each $(\rho_i, \hat{\rho}_j)$-pair, until we fill the matrix $\hat{W}$. We can finally obtain the intermediate variable $\hat{z}$, now as the solution to the linear system with $\hat{W}$ and y,
$$\sum_{k=1}^{N} \hat{W}_{i,k} \hat{z}_k = y_i\,. \tag{16}$$

Again, we can find a unique solution to this linear system because of the well-conditioned requirement of $\hat{W}$ in Definition 1. It is then sufficient to construct the measurement observable $\hat{\mathcal{M}}(\vartheta_y)$ as

$$\hat{\mathcal{M}}(\vartheta_y) = \sum_{k=1}^{N} \hat{z}_k \hat{\rho}_k \,. \tag{17}$$

We clear the relation between ϑ_y and $\hat{z}$ further below, which is crucial in this construction.

The fact that the construction produces the correct outcome for each $i \in [N]$ should not be surprising by now

$$\operatorname{tr}(\rho_i \hat{\mathcal{M}}(\vartheta_y)) = \operatorname{tr}\left(\rho_i \sum_{k=1}^{N} \hat{z}_k \hat{\rho}_k\right) = \sum_{k=1}^{N} \hat{z}_k \operatorname{tr}(\rho_i \hat{\rho}_k) \tag{18}$$

$$= \sum_{k=1}^{N} \hat{z}_k \hat{W}_{i,k} = y_i \,. \tag{19}$$

The question remains whether we can implement $\hat{\mathcal{M}}(\vartheta_y)$ as a PQC since, so far, we have only stated each of the approximated states $\hat{\rho}_j$ can individually be prepared as a PQC. Indeed, we could use classical controls to prepare each of the U_j state-preparation-unitaries, along with extra $\log(N)$ auxiliary qubits.

Suppose we wanted to stay in the spirit of Theorem 1. We could first construct a classical probability distribution from $\hat{z}$ and then sample and prepare the $\hat{\rho}_k$ with probability proportional to $|\hat{z}_k|$, keeping track of potential sign flips. However, potentially, for each different $\hat{\rho}_k$, we would need to run a different circuit U_k. This would give up the PQC picture slightly. Hence we need to reconcile this sampling from the $\hat{z}$-probability distribution as part of the PQC itself, for which we shall use a few extra qubits.

As before, from $\hat{z} = (\hat{z}_1, \ldots, \hat{z}_N)$, we construct the probability distribution $\hat{p}$, and a vector of signs $\hat{s}$, $\hat{s}_j = \operatorname{sign}(\hat{z}_j)$, with

$$\hat{p}_k = \frac{|\hat{z}_k|}{\sum_{j=1}^{N} |\hat{z}_j|} \propto |\hat{z}_k|, \tag{20}$$

so that it holds $\hat{z}_k = \hat{s}_k \hat{p}_k \sum_{j=1}^{N} |\hat{z}_j|$. We initialize a quantum circuit with two registers, the n-qubit input register, and a $\lceil \log_2(N) \rceil$-qubit auxiliary register. First, we perform amplitude encoding $V(\vartheta_y^{(1)})$ for the distribution $\hat{p}$ on the auxiliary register, to get

$$V(\vartheta_y^{(1)})|0\rangle = \sum_{j=1}^{N} \sqrt{\hat{p}_j} |j\rangle \,. \tag{21}$$

For amplitude encoding, we consider the arbitrary state preparation protocol proposed in Ref. [1]. There, with a fixed circuit structure, we find the mapping from the amplitudes to be encoded to the rotation angles to be used in the parametrized circuit. With our notation, this mapping corresponds to $\hat{p} \mapsto \vartheta_y^{(1)}$. The protocol in [1] is efficient in the length of the vector to be encoded. For fixed $\rho_1, \ldots, \rho_N$, the probabilities $\hat{p}$ depend only on y, hence the explicit y-dependence of $\vartheta_y^{(1)}$. We call these parameters $\vartheta_y^{(1)}$ because they are not the only variational parameters that come into play.

Next, for each $k \in [N]$, we construct the controlled rotation CU_k which, if the auxiliary register is in state $|k\rangle$, implements the inverse of the kth state-preparation-unitary $U_k^\dagger$ on the input register, and does nothing if the auxiliary register is in a different state

$$\mathrm{CU_k}\left[\rho \otimes |k'\rangle\langle k'|\right] := \begin{cases} U_k^\dagger \rho U_k \otimes |k\rangle\langle k| & \text{if } k = k' \\ \rho \otimes |k'\rangle\langle k'| & \text{else} \,. \end{cases} \tag{22}$$

We call CU the sequence of all such controlled gates $\mathrm{CU} := \mathrm{CU}_N \ldots \mathrm{CU}_1$. With this, if the auxiliary register is in a single computational-basis state vector $|j\rangle$, the effect of CU is

$$\mathrm{CU}\left[\rho \otimes |j\rangle\langle j|\right] = U_j^\dagger \rho U_j \otimes |j\rangle\langle j| \,. \tag{23}$$

After all the controlled rotations, we perform a product measurement on both registers $\mathcal{O}(\vartheta_y^{(2)}) = \mathcal{O}_{\text{input}} \otimes \mathcal{O}_{\text{aux}}(\vartheta_y^{(2)})$. On the input register, we measure the projector onto the $|0\rangle$ state $\mathcal{O}_{\text{input}} = |0\rangle\langle 0|$. On the auxiliary register, we perform a computational basis measurement with sign flips according to the sign vector $\hat{s}$ to arrive at

$$\mathcal{O}_{\text{aux}}(\vartheta_{\mathrm{y}}^{(2)}) = \sum_{j=1}^{N} \hat{s}_j |j\rangle\langle j| \,. \tag{24}$$

Again, $\hat{s}$ depends on y, so now the variational parameters $\vartheta_y^{(2)}$ are the ones controlling whether the jth computational basis state has a sign-flip $s_j = -1$ or not $s_j = 0$. We can again fix a circuit structure such that each specification of $\hat{s}$ corresponds only to altering the variational parameters $\vartheta_y^{(2)}$. we shall denote $\vartheta_y = (\vartheta_y^{(1)}; \vartheta_y^{(2)})$, uniting both kinds of variational parameters.

Measuring the combined observable on the system while the auxiliary state is in the computational basis state $|k\rangle$ produces the outcome

$$\mathrm{tr}\left[\rho \otimes |k\rangle\langle k| \mathcal{O}(\vartheta^{(2)})\right] = \mathrm{tr}\left[\rho \mathcal{O}_{\text{input}}\right] \mathrm{tr}\left[|k\rangle\langle k| \mathcal{O}_{\text{aux}}(\vartheta^{(2)})\right] = \langle 0|\rho|0\rangle \hat{s}_k \,. \tag{25}$$

Finally, we put everything together to prove the correctness of the construction.

For a given ρ_i, our task is to measure $\mathrm{tr}[\rho_i \hat{\mathcal{M}}(\vartheta_y^{(2)})]$. The computation starts with the input register initialized as ρ_i and the auxiliary register on the $|0\rangle$ state. First, we perform amplitude encoding $V(\vartheta^{(1)})$ on the auxiliary register and then apply the controlled rotation unitary CU, to arrive at

$$\mathrm{CU}\left[\rho_i \otimes V(\vartheta^{(1)})|0\rangle\langle 0|V^\dagger(\vartheta^{(1)})\right] = \sum_{j,j'} \sqrt{\hat{p}_j \hat{p}_{j'}}\, \mathrm{CU}\left[\rho_i \otimes |j\rangle\langle j'|\right] = \sum_{j,j'} \sqrt{\hat{p}_j \hat{p}_{j'}}\, U_j^\dagger \rho_i U_{j'} \otimes |j\rangle\langle j'| \,. \tag{26}$$

Second and last, we measure $\mathcal{O}(\vartheta^{(2)})$ on this state, to get

$$\mathrm{tr}\left[\sum_{j,j'} \sqrt{\hat{p}_j \hat{p}_{j'}} U_j^\dagger \rho_i U_{j'} \otimes |j\rangle\langle j'| \mathcal{O}(\vartheta^{(2)})\right] = \sum_{j,j'} \sqrt{\hat{p}_j \hat{p}_{j'}}\, \mathrm{tr}\left[U_j^\dagger \rho_i U_{j'} \otimes |j\rangle\langle j'| \mathcal{O}_{\text{input}} \otimes \mathcal{O}_{\text{aux}}(\vartheta^{(2)})\right] \tag{27}$$

$$= \sum_{j,j'} \sqrt{\hat{p}_j \hat{p}_{j'}}\, \mathrm{tr}\left[U_j^\dagger \rho_i U_{j'} \mathcal{O}_{\text{input}}\right] \mathrm{tr}\left[|j\rangle\langle j'| \mathcal{O}_{\text{aux}}(\vartheta^{(2)})\right] \,. \tag{28}$$

We only need to rearrange the formulas to get our original statement out, up to a multiplicative factor of $\sum_k |\hat{z}_k|$, to get

$$\sum_{j,j'} \sqrt{\hat{p}_j \hat{p}_{j'}}\, \mathrm{tr}\left[U_j^\dagger \rho_i U_{j'} \mathcal{O}_{\text{input}}\right] \mathrm{tr}\left[|j\rangle\langle j'| \mathcal{O}_{\text{aux}}(\vartheta^{(2)})\right] = \sum_{j,j'} \sqrt{\hat{p}_j \hat{p}_{j'}}\, \mathrm{tr}\left[U_j^\dagger \rho_i U_{j'} |0\rangle\langle 0|\right] \mathrm{tr}\left[|j\rangle\langle j'| \sum_k \hat{s}_k |k\rangle\langle k|\right] \tag{29}$$

$$= \sum_{j,j'} \sqrt{\hat{p}_j \hat{p}_{j'}}\, \mathrm{tr}\left[\rho_i \hat{\rho}_j\right] \hat{s}_j \delta_{j,j'} \tag{30}$$

$$= \sum_j \hat{p}_j \hat{s}_j\, \mathrm{tr}\left[\rho_i \hat{\rho}_j\right] \tag{31}$$

$$= \frac{\sum_j \hat{z}_j\, \mathrm{tr}\left[\rho_i \hat{\rho}_j\right]}{\sum_k |\hat{z}_k|} \tag{32}$$

$$= \frac{\mathrm{tr}\left[\rho_i \hat{\mathcal{M}}_y\right]}{\sum_k |\hat{z}_k|} \,. \tag{33}$$

Indeed, in order to reach our goal, we need to multiply the result of the construction with the 1-norm of the intermediate variable $\hat{z}$. Thus completing the proof that PQCs with fixed structure can learn arbitrary data labelings, provided the input states are distinguishable enough.

[1] M. Mottonen, J. J. Vartiainen, V. Bergholm, and M. M. Salomaa, "Transformation of quantum states using uniformly controlled rotations," arXiv:0407010 (2004).
